# Anterior tumour location as a risk factor for local recurrence after laparoscopic surgery for stage I ultra-low rectal cancers: exploratory analysis of the Ultimate trial

**DOI:** 10.1093/bjsopen/zrag128

**Published:** 2026-09-30

**Authors:** Atsushi Kohyama, Shinobu Ohnuma, Yuichiro Tsukada, Michiaki Unno, Jun Watanabe, Yosuke Fukunaga, Yasumitsu Hirano, Kazuhiro Sakamoto, Hiroki Hamamoto, Masanori Yoshimitsu, Hisanaga Horie, Nobuhisa Matsuhashi, Yoshiaki Kuriu, Shuntaro Nagai, Madoka Hamada, Shinichi Yoshioka, Tamuro Hayama, Koki Otsuka, Yusuke Inoue, Kazuki Ueda, Yuji Toiyama, Satoshi Maruyama, Shigeki Yamaguchi, Keitaro Tanaka, Motoko Suzuki, Toshihiro Misumi, Takeshi Naitoh, Masahiko Watanabe, Masaaki Ito, Ultimate Trial Group in the Japan Society of Laparoscopic Colorectal Surgery, Shinobu Ohnuma, Yuichiro Tsukada, Masaaki Ito, Motoko Suzuki, Toshihiro Misumi, Jun Watanabe, Yosuke Fukunaga, Yasumitsu Hirano, Kazuhiro Sakamoto, Hiroki Hamamoto, Keitaro Tanaka, Masanori Yoshimitsu, Hisanaga Horie, Nobuhisa Matsuhashi, Yoshiaki Kuriu, Shuntaro Nagai, Madoka Hamada, Shinichi Yoshioka, Tamuro Hayama, Koki Otsuka, Yusuke Inoue, Kazuki Ueda, Yuji Toiyama, Satoshi Maruyama, Shigeki Yamaguchi, Takeshi Naitoh, Masahiko Watanabe

**Affiliations:** Department of Surgery, Tohoku University Graduate School of Medicine, Sendai, Japan; Department of Surgery, Iwaki City Medical Center, Iwaki, Japan; Department of Surgery, Tohoku University Graduate School of Medicine, Sendai, Japan; Department of Colorectal Surgery, National Cancer Center Hospital East, Chiba, Japan; Department of Surgery, Tohoku University Graduate School of Medicine, Sendai, Japan; Department of Surgery, Gastroenterological Center, Yokohama City University Medical Center, Yokohama, Japan; Department of Colorectal Surgery, Kansai Medical University, Osaka, Japan; Department of Gastroenterological Surgery, Cancer Institute Hospital, Japanese Foundation of Cancer Research, Tokyo, Japan; Department of Colorectal Surgery, Kansai Medical University Medical Center, Osaka, Japan; Department of Gastroenterological Surgery, Saitama Medical University International Medical Center, Hidaka, Japan; Department of Coloproctological Surgery, Juntendo University Faculty of Medicine, Tokyo, Japan; Department of General and Gastroenterological Surgery, Osaka Medical and Pharmaceutical University, Osaka, Japan; Department of Surgery, Hiroshima City North Medical Center Asa Citizens Hospital, Hiroshima, Japan; Department of Surgery, Hiroshima City Hiroshima Citizens Hospital, Hiroshima, Japan; Department of Surgery, Jichi Medical University, Tochigi, Japan; Department of Gastroenterological Surgery and Pediatric Surgery, Graduate School of Medicine, Gifu University, Gifu, Japan; Department of Surgery, Kyoto Prefectural University of Medicine, Kyoto, Japan; Department of Surgery and Oncology, Graduate School of Medical Sciences, Kyushu University, Fukuoka, Japan; Department of Gastrointestinal Surgery, Kansai Medical University Hospital, Hirakata, Japan; Department of Surgery, Yao Municipal Hospital, Osaka, Japan; Department of Surgery, Teikyo University School of Medicine, Tokyo, Japan; Department of Surgery, Iwate Medical University School of Medicine, Iwate, Japan; Department of Surgery, Graduate School of Biomedical Sciences, Nagasaki University, Nagasaki, Japan; Division of Endoscopic and Colorectal Surgery, Department of Surgery, Kindai University Faculty of Medicine, Osaka, Japan; Department of Gastrointestinal and Pediatric Surgery, Mie University, Tsu, Japan; Department of Gastroenterological Surgery, Niigata Cancer Center Hospital, Niigata, Japan; Division of Colorectal Surgery, Department of Surgery, Tokyo Women's Medical University, Tokyo, Japan; Department of General and Gastroenterological Surgery, Osaka Medical and Pharmaceutical University, Osaka, Japan; Department of General, Breast and Digestive Surgery, Otsu City Hospital, Otsu, Japan; Department of Data Science, National Cancer Center Hospital East, Chiba, Japan; Department of Data Science, National Cancer Center Hospital East, Chiba, Japan; Department of Lower Gastrointestinal Surgery, Kitasato University School of Medicine, Kanagawa, Japan; Department of Lower Gastrointestinal Surgery, Kitasato University School of Medicine, Kanagawa, Japan; Department of Colorectal Surgery, National Cancer Center Hospital East, Chiba, Japan

**Keywords:** total mesorectal excision, intersphincteric resection, low anterior resection, abdominoperineal resection, resection margin

## Abstract

**Background:**

Anterior tumour location is an established risk factor for local recurrence (LR) in advanced rectal cancer, but its significance in stage I ultra-low rectal cancer remains unclear.

**Methods:**

This exploratory supplemental analysis used data from Ultimate trial, a multicentre prospective phase II study across 47 Japanese institutions (2014–2017). Patients with cT1–2 N0 M0 ultra-low rectal cancer (≤ 5 cm from the anal verge) underwent laparoscopic resection. Tumour location was categorized as anterior (10–2 o’clock) or non-anterior (2–10 o’clock). The primary endpoint was LR; secondary endpoints included recurrence-free survival, overall survival, and stoma-free survival.

**Results:**

Of 300 enrolled patients, 297 were analysed (median follow-up 37.2 months); 93 tumours (31.3%) were anterior. Baseline characteristics, operative outcomes, and pathological findings were similar between the two groups. The 3-year cumulative LR rate was 11.9% for anterior tumours and 3.0% for non-anterior tumours (hazard ratio (HR) 4.46; 95% confidence interval (c.i.) 1.67 to 11.89; *P* = 0.001). Multivariable analysis showed an independent association between anterior location and LR (HR 4.68; 95% c.i. 1.72 to 12.74); subgroup analysis revealed no difference in clinical (c) T1 patients, but a significantly higher LR rate for anterior tumours in cT2 patients (25.3% *versus* 4.7%; *P* < 0.001). Three-year recurrence-free survival (83.9% for anterior tumours *versus* 88.9% for non-anterior tumours; *P* = 0.222), overall survival (> 95%), and stoma-free survival were comparable between the two groups.

**Conclusion:**

Anterior tumour location in the ultra-low rectum was associated with an increased risk of LR, particularly in cT2 patients, highlighting the need for a more refined treatment strategy and vigilant monitoring.

## Teaser

This exploratory analysis of a prospective multicentre phase II trial evaluated whether anterior tumour location increases the risk of local recurrence in clinical stage I ultra-low rectal cancer. Among 297 patients, anterior tumours showed a significantly higher 3-year local recurrence rate, particularly in clinical T2 disease, whereas survival outcomes were comparable between groups. These findings suggest that anterior tumour location may represent a clinically relevant risk factor, warranting further investigation and careful postoperative surveillance.

## Introduction

Laparoscopic surgery is a widely accepted minimally invasive approach for rectal cancer. Randomized clinical trials^1–3^, including the COLOR II trial^4^, have demonstrated that laparoscopic rectal surgery achieves equivalent safety, resection margins, and oncological outcomes to open surgery. The Ultimate trial^5^ recently reported favourable long-term outcomes, high anus-preservation rates, and satisfactory postoperative anal function following laparoscopic resection for clinical (c) T1/T2 ultra-low rectal cancers located within 5 cm of the anal verge (AV). For such early-stage tumours, preoperative chemoradiotherapy is generally not indicated, and total mesorectal excision remains the standard curative procedure.

However, despite technical advances and improved visualization, local recurrence (LR) after curative resection remains a major clinical concern. Reported LR rates after laparoscopic rectal cancer surgery range from 5% to 12% in randomized trials^6–8^, and remain at approximately 4% even among patients with pathological (p) T1/T2 disease who undergo local excision (LE) followed by completion total mesorectal excision^9^. Accordingly, neither neoadjuvant radiotherapy nor chemoradiotherapy is used in the management of early-stage rectal cancer.

Tumour location within the rectum is an important determinant of recurrence. Lower rectal cancers exhibit higher LR rates than upper or middle rectal tumours^10–12^, and emerging evidence suggests that the circumferential orientation within the rectum further influences oncological outcomes. Anteriorly situated tumours are associated with higher LR and poorer survival^10,13^, possibly due to the thin anterior mesorectal tissue and the close proximity of the prostate, urethra, or vagina, which increases the likelihood of a positive circumferential resection margin (CRM) or incomplete mesorectal excision. Most existing data are from patients with advanced disease treated with multimodality therapy^10–13^, but and the relevance of these findings to early-stage rectal cancer is unclear.

The present study investigated whether anterior tumour location represents an independent risk factor for LR in patients with cT1/T2 stage I ultra-low rectal cancer undergoing laparoscopic total mesorectal excision.

## Methods

### Study design and participants

This was an exploratory supplemental analysis of the Ultimate trial evaluating the efficacy and safety of laparoscopic surgery for ultra-low rectal cancer. Patients were recruited from 47 specialized institutes in Japan between January 2014 and March 2017, with follow-up continuing until 2021^5^. This study was registered with the University Hospital Medical Information Network (UMIN) Clinical Trials Registry (UMIN000011750).

Eligible patients were adults (≥ 20 years) with histologically confirmed rectal cancer located within 5 cm of the AV or within 3 cm of the dentate line (DL), clinically staged as T1–2 N0 M0. Clinical staging was determined by colonoscopy, chest–abdominal–pelvic computed tomography with slice thickness < 10 mm, trans-anal ultrasonography, or magnetic resonance imaging. Patients who were not candidates for LE or who required additional surgery with lymphadenectomy after LE were included in the study. The exclusion criteria included having another malignancy within the past 5 years, conversion to open surgery before resection, intraoperative detection of distant metastasis, inadequate follow-up, and receiving neoadjuvant chemotherapy or chemoradiotherapy.

Tumour location, determined by rigid rectoscopy, endoscopy, or digital rectal examination, was classified before surgery according to the area of deepest infiltration: tumours between the 10 and 2 o’clock positions were defined as anterior, whereas those between the 2 and 10 o’clock positions were defined as non-anterior. Tumour distance from the AV and DL was determined by the same method both before and during surgery.

### Registration

All eligible patients who met the inclusion and exclusion criteria were registered before surgery by calling the registration office at the National Cancer Center Hospital East (Chiba, Japan). An eligibility report form was sent to the data centre at the clinical trial coordinating office at the National Cancer Center Hospital East.

### Quality control, procedures, and follow-up

Only experienced surgeons who had performed more than 30 laparoscopic and 30 open operations for rectal carcinoma were accredited by the study chair to participate in this study. A centralized photographic review of the surgical procedure was performed for all patients. This included one image of the pelvic floor after total mesorectal excision and, in patients undergoing intersphincteric resection (ISR), an additional image documenting the level of the mucosal incision during the transanal phase^5^. Laparoscopic procedures included laparoscopic low anterior resection (LAR), ISR, Hartmann surgery, and abdominoperineal resection (APR). All surgical procedures were performed as described previously^14,15^. Laparoscopic dissection was performed down to approximately the lower two-thirds level of the prostate or vagina. When further dissection was required during ISR or APR, it was performed via a perineal approach under direct visualization. Briefly, laparoscopic resection of the rectum with adequate lymphadenectomy was performed. The extent of the lymphadenectomy, the ligation site, and division of the inferior mesenteric vessels were determined by the surgeon. Rectal mobilization, mesorectal excision, rectal transection, specimen removal, and reconstruction were performed using a pneumoperitoneal or extracorporeal approach via a < 8-cm incision. Bowel anastomoses were performed intracorporeally via a small incision using the double-stapling technique or transanal hand-sewn sutures^5^. To assess potential institutional differences, hospitals with more than 11 registered patients were classified as high-volume centres, and those with ten or fewer patients were classified as low-volume centres; treatment outcomes were compared between the two groups.

For sphincter-preserving operations, the decision to perform a protective ileostomy was based on the surgeon’s technical evaluation of anastomosis quality. Faecal diversion refers to a temporary diverting stoma created after sphincter-preserving surgery. Stoma creation at initial surgery refers to any stoma constructed during the index operation, including both temporary diverting stomas and permanent stomas associated with APR or Hartmann’s surgery. Conversion was defined as requiring an abdominal incision exceeding 8 cm to manage intraoperative complications or tumour extension. Surgical methods and pathology results were recorded according to the Japanese Classification of Colon and Rectal Carcinoma (seventh edition)^16^ and the TNM classification (sixth edition)^17^. A macroscopically adequate dissection margin was defined as the absence of gross tumour exposure along the dissection plane, as assessed intraoperatively by the operating surgeon. A pathological resection margin was classified as R0 when no tumour exposure was identified pathologically and as R1 when tumour exposure was identified along any resection margin.

For postoperative surveillance, blood tests, including carcinoembryonic antigen (CEA) and carbohydrate antigen 19-9 (CA19-9), were performed every 3 months; chest–abdominal–pelvic computed tomography scans were obtained every 6 months; and a colonoscopy was conducted at 1, 3, and 5 years after surgery. LR was defined as tumour regrowth within the pelvis.

### Outcomes of interest

The primary aim of this study was to determine whether anterior tumour location is an independent risk factor for LR after laparoscopic surgery in patients with stage I ultra-low rectal cancer. Secondary endpoints were 3-year recurrence-free survival (RFS), overall survival (OS), and stoma-free rates between anterior and non-anterior tumours and in subgroup analyses according to cT category.

### Statistical analysis

All analyses were performed using JMP Pro version 16.0 (SAS Institute, Cary, NC, USA). Continuous variables (age, body mass index, tumour size, distance from the AV/DL, CEA, CA19-9, operation time, blood loss, and length of hospital stay) are expressed as the median with range (minimum–maximum) and were compared using the Mann–Whitney *U* test. Follow-up duration is reported as the median with interquartile range (i.q.r.). Categorical variables are expressed as counts and percentages and were compared using Pearson’s χ^2^ test or Fisher’s exact test.

LR, RFS, and OS were estimated by the Kaplan–Meier method, and the significance of differences between groups was assessed using log-rank tests. Two-sided *P* < 0.05 was considered statistically significant. Univariable Cox proportional hazards regression was first performed to identify factors associated with LR. Variables with *P* < 0.10 and those considered clinically relevant were entered into the multivariable Cox model, while also taking into account the limited number of events to maintain an appropriate events-per-variable ratio. The proportional hazards assumption was evaluated by testing time-dependent covariates (interaction terms between each variable and survival time) and by visually assessing Kaplan–Meier survival curves for evidence of non-proportionality. Hazard ratios (HRs) and their 95% confidence interval (c.i.) were calculated. Continuous variables were analysed per 1-unit increase.

## Results

### Study population

In all, 300 patients were enrolled across 47 Japanese Society for Cancer of the Colon and Rectum-affiliated institutions. Of these patients, three were excluded, one because of conversion to open surgery before the operation, one with intraoperative liver metastasis, and one with no available follow-up data (*Fig. S1*), leaving 297 patients for analysis. The median follow-up duration was 37.2 (i.q.r., 36.2–39.5) months.

Baseline demographics and clinical characteristics are presented in *Table 1*. In all, 93 tumours (31.3%) were classified as anterior and 204 (68.7%) were classified as non-anterior. The anterior and non-anterior groups did not differ significantly in terms of age (*P* = 0.210), sex distribution (*P* = 1.000), cT category (*P* = 1.000), tumour size (*P* = 0.344), tumour distance from the AV and DL, morphology, serum tumour markers, LE before surgery, or histological type.

**Table 1:** Demographics and baseline clinical characteristics of all patients and for those with anterior and non-anterior tumours separately

	Total (*n* = 297)	Anterior (*n* = 93)	Non-anterior (*n* = 204)	P*
Age (years), median (range)	65 (30–87)	66 (30–87)	64.5 (36–87)	0.210†
**Sex**				1.000
Female	112 (37.7%)	35 (37.6%)	77 (37.8%)	
Male	185 (62.3%)	58 (62.4%)	127 (62.3%)	
BMI (kg/m^2^), median (range)	22.7 (15.9–38.6)	22.7 (17.2–38.6)	22.7 (15.9–34.0)	0.599†
**ECOG PS**				0.241
0	274 (92.3%)	83 (89.3%)	191 (93.6%)	
1	23 (7.7%)	10 (10.8%)	13 (6.4%)	
**Clinical T category**				1.000
T1	169 (56.9%)	53 (57.0%)	116 (56.9%)	
T2	128 (43.1%)	40 (43.0%)	88 (43.1%)	
Tumour size (mm), median (range)	25 (0–85)	22 (0–65)	25 (0–85)	0.344†
AV (mm), median (range)	40 (0–55)	45 (5–50)	40 (0–55)	0.870†
DL (mm), median (range)	20 (−15, 40)	20 (−15, 40)	20 (−15, 35)	0.738†
**Morphology**				0.354
Ulcerative	98 (33.0%)	27 (29.0%)	71 (34.8%)	
Others	199 (67.0%)	66 (71.0%)	133 (65.2%)	
CEA (ng/ml), median (range)	2.1 (0.4–27.0)	2.3 (0.5–12.7)	2.0 (0.4–27.0)	0.076†
CA19-9 (U/ml), median (range)	8.0 (0.1–244.0)	7.9 (0.1–52.0)	8.0 (0.1–244.0)	0.362†
**LE before surgery**				0.487
Yes	82 (27.6%)	23 (24.7%)	59 (28.9%)	
No	215 (72.4%)	70 (75.3%)	145 (71.1%)	
**Histological type**				0.711‡
Well/moderately differentiated	284 (95.6%)	88 (94.6%)	196 (96.1%)	
Poorly differentiated/mucinous	9 (3.0%)	3 (3.2%)	6 (2.9%)	
Papillary	4 (1.4%)	2 (2.2%)	2 (1.0%)	

Values are *n* (%) unless otherwise stated. BMI, body mass index; ECOG PS, Eastern Cooperative Oncology Group performance status; AV, anal verge; DL, dentate line; CEA, carcinoembryonic antigen; CA19-9, carbohydrate antigen 19-9; LE, local excision. *Fisher’s exact test, except †Mann–Whitney *U* test and ‡Pearson’s χ^2^ test.

### Surgical and pathological outcomes

Surgical and pathological outcomes are summarized in *Table 2*. The distribution of surgical procedures (LAR, ISR, APR, Hartmann) was comparable between the anterior and non-anterior groups. Surgical outcomes, including the rate of macroscopically adequate dissection margin (*P* = 1.000), distal margin (*P* = 0.633), anastomotic leakage (*P* = 0.654), and stoma creation at initial surgery (*P* = 0.503), were also similar between the two groups. Faecal diversion was performed in 84.9% and 84.3% of patients in the anterior and non-anterior groups, respectively (*P* = 1.000). At final follow-up, the proportion of patients who were stoma-free did not differ significantly between the anterior and non-anterior groups (*P* = 0.841). Pathological findings, including T categories (*P* = 0.577), nodal status (*P* = 1.000), pathological stage (*P* = 0.864), and pathological resection margin (*P* = 1.000), did not differ significantly between the two groups. The rates of adjuvant chemotherapy were also comparable between the anterior and non-anterior groups (*P* = 0.501).

**Table 2:** Surgical and pathological outcomes for all tumours and according to anterior and non-anterior tumour location separately

	Total (*n* = 297)	Anterior (*n* = 93)	Non-anterior (*n* = 204)	P*
Operative time (min), median (range)	286 (96–656)	295 (114–614)	285 (96–656)	0.884†
Blood loss (ml), median (range)	34 (0–740)	30 (0–740)	35 (0–564)	0.763†
**Surgical procedure**				
LAR	105 (35.4%)	27 (29.0%)	78 (38.2%)	0.150
ISR	171 (57.6%)	58 (62.4%)	113 (55.6%)	0.311
APR	19 (6.4%)	7 (7.5%)	12 (5.9%)	0.614
Hartmann	2 (0.7%)	1 (1.1%)	1 (0.5%)	0.529
**Macroscopically adequate dissection margin**				1.000
Yes	297 (100.0%)	93 (100.0%)	204 (100.0%)	
No	0	0	0	
Distal margin (mm), median (range)	15 (0.3–87)	14 (0.3–87)	15 (1.2–65)	0.633
**Anastomotic leakage**				
All grades	25 (8.4%)	9 (9.7%)	16 (7.8%)	0.654
Grade ≥ 3	17 (5.7%)	5 (5.4%)	12 (5.9%)	1.000
**Stoma creation at initial surgery**				0.503
Yes	272 (91.6%)	87 (93.6%)	185 (90.7%)	
No	25 (8.4%)	6 (6.5%)	19 (9.3%)	
Faecal diversion	251 (84.5%)	79 (84.9%)	172 (84.3%)	1.000
LOS (days), median (range)	14 (6–391)	15 (6–380)	14 (6–391)	0.815†
Mortality within 30-days	0	0	0	
Stoma-free at final follow-up	265 (89.2%)	84 (90.3%)	181 (88.7%)	0.841
**Adjuvant chemotherapy**				0.501
Yes	48 (16.2%)	17 (18.3%)	31 (15.2%)	
No	249 (83.8%)	76 (81.7%)	173 (84.8%)	
**Pathological T category**				0.577‡
Tis	5 (1.7%)	1 (1.1%)	4 (2.0%)	
T1	157 (52.9%)	48 (51.6%)	109 (53.4%)	
T2	105 (35.4%)	37 (39.8%)	68 (33.3%)	
T3	30 (10.1%)	7 (7.5%)	23 (11.3%)	
**Pathological N category**				1.000
N–	249 (83.8%)	78 (83.9%)	171 (83.8%)	
N+	48 (16.2%)	15 (16.1%)	33 (16.2%)	
**Pathological stage**				0.864‡
0	5 (1.7%)	1 (1.1%)	4 (2.0%)	
I	224 (75.4%)	72 (77.4%)	152 (74.5%)	
II	20 (6.7%)	5 (5.4%)	15 (7.4%)	
III	48 (16.2%)	15 (16.1%)	33 (16.2%)	
**Pathological resection margin**				1.000
R0	297 (100.0%)	93 (100.0%)	204 (100.0%)	
R1	0	0	0	
**Vascular invasion**				0.368‡
Yes	169 (56.9%)	58 (62.4%)	111 (54.4%)	
No	127 (42.8%)	35 (37.6%)	92 (45.1%)	
Unknown	1 (0.3%)	0 (0.0%)	1 (0.5%)	
**Lymphatic invasion**				0.547‡
Yes	119 (40.1%)	34 (36.6%)	85 (41.7%)	
No	177 (59.6%)	59 (63.4%)	118 (57.8%)	
Unknown	1 (0.3%)	0 (0.0%)	1 (0.5%)	
**Budding grade**				0.249‡
Low	122 (41.1%)	41 (44.1%)	81 (39.7%)	
Intermediate	32 (10.8%)	5 (5.4%)	27 (13.2%)	
High	18 (6.1%)	6 (6.5%)	12 (5.9%)	
Unknown	125 (42.1%)	41 (44.1%)	84 (41.2%)	

Values are *n* (%) unless otherwise stated. min, minutes; LAR, low anterior resection; ISR, intersphincteric resection; APR, abdominoperineal resection; LOS, length of hospital stay. *Fisher’s exact test, except †Mann–Whitney *U* test and ‡Pearson’s χ^2^ test.

### Oncological outcomes

The 3-year cumulative LR rate for the entire cohort was 5.9% (95% c.i. 3.7 to 9.3). Stratified by tumour location, LR was significantly higher for anterior than non-anterior tumours (HR 4.46; 95% c.i. 1.64 to 12.11; *P* = 0.001; *Fig. 1*). There was no significant difference between anterior and non-anterior tumours in the 3-year RFS (HR 1.50; 95% c.i. 0.75 to 3.01; *P* = 0.222) and OS (HR 0.57; 95% c.i., 0.19 to 1.74; *P* = 0.377; *Fig. 1*). No significant differences in LR were detected between high-volume and low-volume centres, either in the overall cohort (*Fig. S2*) or among patients with anterior lesions (*Fig. S2*).

**Figure 1:**
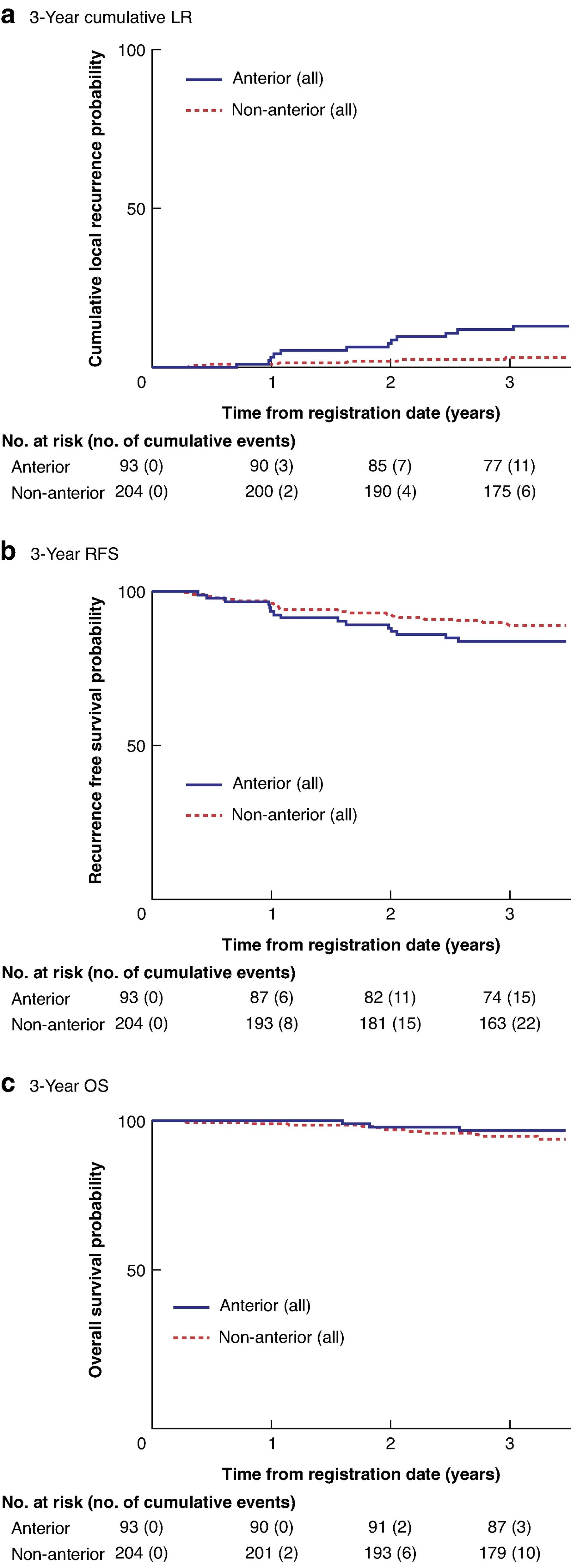
Kaplan–Meier curves of cumulative LR, RFS, and OS stratified by tumour location **a** The 3-year cumulative LR rate for anterior and non-anterior tumours was 11.9% (95% c.i. 6.7% to 20.2%) and 3.0% (95% c.i. 1.4% to 6.6%), respectively; Anterior tumours were associated with a significantly higher risk of LR (HR 4.46; 95% c.i. 1.64 to 12.11; *P* = 0.001). **b** Three-year RFS for anterior and non-anterior tumours was 83.9% (95% c.i. 75.0% to 90.0%) and 88.9% (95% c.i. 83.7% to 92.6%), respectively. There was no significant difference in RFS between anterior and non-anterior tumours (HR 1.50; 95% c.i. 0.75 to 3.01; *P* = 0.222). **c** Three-year OS for anterior and non-anterior tumours was 96.8% (95% c.i. 90.5% to 99.0%) and 95.0% (95% c.i. 91.0% to 97.3%), respectively. There was no significant difference in OS were detected between anterior and non-anterior tumours (HR 0.57; 95% c.i. 0.19 to 1.74; *P* = 0.377). LR, local recurrence; RFS, recurrence-free survival; OS, overall survival; c.i., confidence interval; HR, hazard ratio.

### Risk factors for local recurrence

To identify risk factors for LR, Cox proportional hazards regression analyses were performed that included patient-related variables and surgical factors, such as operative approach and distal resection margin (*Table 3*). In the univariable analysis, cT category (*P* = 0.005), morphology (*P* = 0.002), and tumour location (anterior *versus* non-anterior; *P* = 0.003) emerged as significant predictors of LR. In the multivariable analysis, only tumour location remained an independent predictor (HR 4.68; 95% c.i., 1.72 to 12.74; *P* = 0.003). The interaction term between anterior tumour location and time was statistically significant, suggesting a violation of the proportional hazard assumption for tumour location. To further identify high-risk subsets among anterior lesions, an additional subgroup analyses were performed by cT category (*Fig. 2*). Among patients with cT1 tumours, the 3-year LR rates were low and comparable between anterior and non-anterior tumours (HR 2.12; 95% c.i. 0.26 to 17.27; *P* = 0.443; *Fig. 2a*). In contrast, among patients with cT2 tumours, anterior location was associated with a significantly higher risk of LR (HR 5.89; 95% c.i. 1.87 to 18.51; *P*  *<* 0.001; *Fig. 2b*). The distribution of pT categories among cT2 anterior and non-anterior tumours did not differ significantly between groups (*P* = 0.838; *Table S1*).

**Figure 2:**
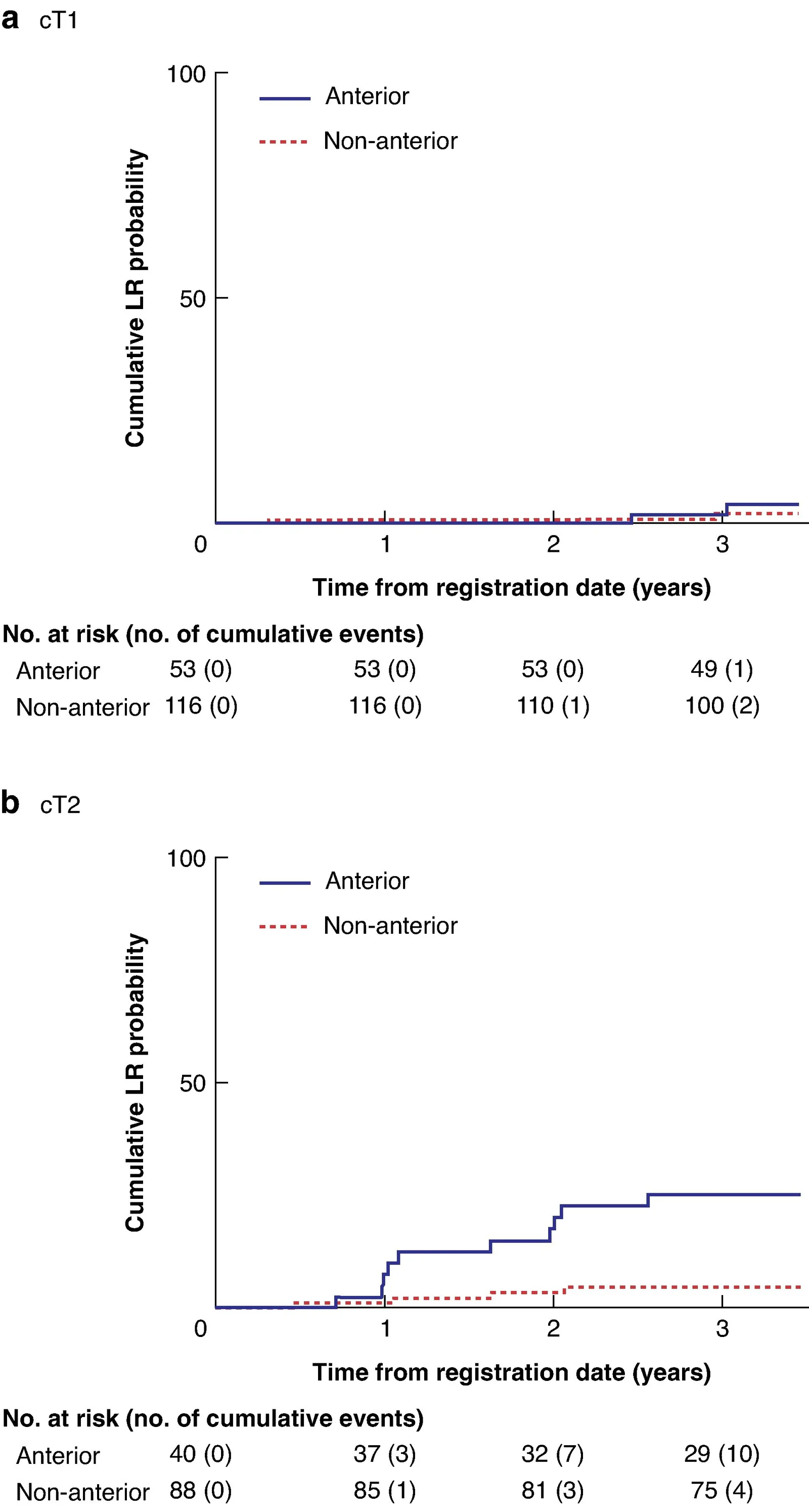
Kaplan–Meier curves of cumulative LR stratified by tumour location according to clinical stage **a** In patients with cT1 disease, the 3-year cumulative LR rate for anterior and non-anterior tumours was 1.9% (95% c.i. 0.3% to 12.2%) and 1.8% (95% c.i. 0.5% to 7.0%), respectively. There was no significant difference in LR rates between anterior and non-anterior tumours (HR 2.12; 95% c.i. 0.26 to 17.27; *P* = 0.443). **b** In patients with cT2 disease, the 3-year cumulative LR rate for anterior and non-anterior tumours was 25.3% (95% c.i. 14.2% to 41.0%) and 4.7% (95% c.i. 1.8% to 11.9%), respectively. In this group, anterior tumours were associated with a significantly higher risk of LR (HR 5.89; 95% c.i. 1.87 to 18.51; *P* < 0.001). LR, local recurrence; c.i., confidence interval; HR, hazard ratio.

**Table 3:** Univariable and multivariable Cox regression analyses of factors associated with local recurrence

	Univariable		Multivariable	
	Hazard ratio	*P*	Hazard ratio	*P*
Age (per 1-year increase)	1.02 (0.98, 1.07)	0.452		
Sex (men *versus* women)	1.24 (0.47, 3.32)	0.662		
BMI (per 1-kg/m^2^ increase)	0.97 (0.83, 1.11)	0.657		
Tumour size (per 1-mm increase)	1.03 (1.00, 1.06)	0.055	1.02 (0.98, 1.06)	0.250
Clinical T category (cT2 *versus* cT1)	4.97 (1.63, 15.09)	0.005	2.35 (0.53, 10.49)	0.264
Morphology (ulcerative *versus* others)	2.89 (1.49, 5.61)	0.002	1.47 (0.43, 5.01)	0.535
Histological type (poorly differentiated/mucinous *versus* others)	2.10 (0.28, 15.78)	0.471		
Location (anterior *versus* non-anterior)	4.45 (1.67, 11.87)	0.003	4.68 (1.72, 12.74)	0.003
Location from AV (per 1-mm increase)	0.97 (0.93, 1.01)	0.100	0.98 (0.94, 1.03)	0.417
CEA (high *versus* normal)	0.84 (0.19, 3.63)	0.810		
CA19-9 (high *versus* normal)	1.98 (0.26, 14.92)	0.505		
Distal margin (per 1-mm increase)	0.98 (0.92, 1.03)	0.490		
LAR (*versus* others)	1.09 (0.85, 1.38)	0.500		
ISR (*versus* others)	0.90 (0.36, 2.29)	0.827		
APR (*versus* others)	3.21 (0.93, 11.10)	0.065	1.20 (0.22, 6.50)	0.829
LE before surgery (yes *versus* no)	0.15 (0.02, 1.11)	0.063	0.41 (0.05, 3.72)	0.428

Values in parentheses are 95% confidence intervals. BMI, body mass index; AV, anal verge; CEA, carcinoembryonic antigen; CA19-9, carbohydrate antigen 19-9; LAR, low anterior resection; ISR, intersphincteric resection; APR, abdominoperineal resection; LE, local excision.

## Discussion

This prospective multicentre study revealed that anterior tumour location is associated with an increased risk of LR, even in early-stage (Union for International Cancer Control stage I) lower rectal cancer. Among 297 patients undergoing curative resection at 47 specialized centres in Japan, LR rates were significantly higher for anterior lesions than for non-anterior lesions, particularly in patients with cT2 disease.

Previous studies emphasized the prognostic relevance of tumour location in advanced rectal cancer^10,13^. Franz *et al*.^10^ identified ventral tumours ≥ 4 cm from the AV as independent predictors of LR in stage II/III disease, whereas Nagtegaal *et al*.^13^ found that an anterior location was associated with poorer local control and survival. However, these studies focused on advanced disease. The present study extend this evidence by demonstrating that anterior tumour location is also associated with increased LR risk in stage I ultra-low rectal cancers. Given that LR is strongly influenced by the CRM, this association warrants further investigation. Previous studies demonstrated that a positive or close CRM significantly elevates the LR risk^18,19^, and that anterior tumours are more frequently associated with CRM involvement^20,21^. In the present cohort, anterior tumours exhibited particularly high LR rates in subgroups with deeper invasion (for example, T2), suggesting that achieving an adequate CRM may be especially challenging in these patients. However, detailed CRM distances were not prospectively or systematically recorded in the original study protocol and complete quantitative CRM data were unavailable. Therefore, although anterior tumour location may plausibly relate to CRM adequacy, the present findings cannot establish a causal relationship, and the interpretation should remain cautious. Where available, qualitative CRM status (involved *versus* not involved) is reported.

From a technical perspective, anterior dissection is inherently more challenging due to both the pelvic anatomy and structural features of the mesorectum^22^. Although posterior dissection follows a relatively clear retrorectal plane, the anterior aspect offers a limited surgical margin. The mesorectum is thick posteriorly and laterally, but thinner anteriorly^23^, making secure CRM more difficult. Moreover, the anterior rectum lies close to critical structures, including the prostate, seminal vesicles, urethra, and vagina, thereby restricting the extent of dissection. Tsukada *et al*.^24^ further reported that longitudinal smooth muscle attachment is longer in the anterolateral portion of the anal canal and shorter posteriorly, with thicker smooth muscle layers posteriorly continuous with the levator ani. These anatomical features contribute to the vulnerability of the anterior dissection plane. Sex-related pelvic anatomy also influences surgical complexity. In men, the narrow pelvis and adjacency of the rectum to the prostate and seminal vesicles render anterior dissection particularly difficult. In women, although the rectum is separated from the vagina by a thinner septum, dissection remains constrained. Nagtegaal *et al*.^13^ reported that anterior tumours are more technically demanding than posterior tumours, particularly in men. However, in the present study, LR rates did not differ significantly between sexes, suggesting that anatomical complexity contributes to, but does not fully explain, the higher recurrence risk. Lower rectal cancer has a higher risk of LR than upper or middle rectal cancer. Because all tumours in the present cohort were located in the ultra-low rectum around the anal verge, the anatomical constraints inherent to this distal region applied uniformly across the study population. This region is associated with higher rates of CRM involvement and greater technical difficulty. A meta-analysis reported that the LR rates of rectal cancer after LE followed by complete total mesorectal excision were 4.1% (range 1.7% to 9.4%) for high-risk pT1 tumours and 4% (range 1% to 13%) for pT2 tumours^9^. The LR rates observed in the present study were slightly higher than those reported in this meta-analysis, likely because the cohort consisted exclusively of ultra-low rectal cancers around the anal verge. Distal tumours are known to have higher rates of CRM involvement than more proximal tumours^20,21^. Consistent with this, the positive margin rate in low T2 tumours (11.4%) has been reported to be more than twice that observed in higher T2 tumours^12^, and higher LR rates after APR than after LAR have also been described^10^. For stage I lower anterior rectal cancer, strategies to improve local control warrant further investigation. Robotic surgery, as demonstrated in the REAL trial, has been associated with favourable LR rates compared with laparoscopy^25^ and may offer technical advantages in anatomically constrained anterior dissections. Similarly, neoadjuvant radiotherapy or chemoradiotherapy reduces LR in advanced disease^26–28^, although its potential role in selected high-risk early-stage anterior tumours remains uncertain. Further prospective studies are needed to determine whether these approaches could improve local control in this subgroup.

This study has several limitations. First, although data collection was prospective, the original protocol was not designed to compare anterior and non-anterior tumours, resulting in a limited sample size and wide confidence intervals (HR 4.68; 95% c.i. 1.72 to 12.74). Although the proportional hazards assumption was not fully satisfied, the Cox model was retained because the violation was modest and the HR can still be interpreted as an average effect over time, which is considered reasonable in clinical studies with limited events. Second, the median follow-up of 3 years is relatively short for detecting LR. However, because a substantial proportion of recurrences after curative resection for colorectal cancer occur within the first few years after surgery^29^, the available follow-up still captures the early recurrence pattern in this cohort. Third, some variables were not uniformly documented across participating institutions. CRM distance was not consistently recorded, limiting mechanistic interpretation of the findings. In addition, magnetic resonance imaging was not performed in all patients, mainly because many tumours were early stage tumours. Although magnetic resonance imaging is valuable for assessing depth and localization^30^, tumours near the anal verge can generally be localized reliably using rigid rectoscopy, digital rectal examination, and endoscopy. However, because this was a multicentre study, formal assessment of interobserver reproducibility was not performed, and some degree of misclassification cannot be excluded. Fourth, surgeon experience may have influenced the outcomes. The Endoscopic Surgical Skill Qualification System (ESSQS), established by the Japan Society for Endoscopic Surgery in 2004^31^ ensures high technical standards in laparoscopic surgery^32,33^, and ESSQS-certified surgeons participated in almost all procedures included in this study. In addition, LR rates did not differ significantly between the high-volume and low-volume centres, indicating that surgeon experience and institutional volume were unlikely to have materially influenced the main results. Despite these limitations, the multicentre design and substantial sample size of the present study strengthen the validity of the finding that anterior ultra-low rectal cancers are associated with an increased risk of LR following laparoscopic surgery.

In conclusion, anterior tumour location in ultra-low rectal cancer is associated with an increased risk of LR. Therefore, even early-stage anterior wall lesions may require careful therapeutic planning and close postoperative surveillance.

## Collaborators

Members of the Ultimate Trial Group in the Japan Society of Laparoscopic Colorectal Surgery group are: Shinobu Ohnuma (Tohoku University Graduate School of Medicine, Sendai, Japan); Yuichiro Tsukada, Masaaki Ito, Motoko Suzuki, Toshihiro Misumi (National Cancer Center Hospital East, Chiba, Japan); Jun Watanabe (Yokohama City University Medical Center, Yokohama, Japan); Yosuke Fukunaga (Cancer Institute Hospital, Japanese Foundation of Cancer Research, Tokyo, Japan); Yasumitsu Hirano (Saitama Medical University International Medical Center, Hidaka, Japan); Kazuhiro Sakamoto (Juntendo University Faculty of Medicine, Tokyo, Japan); Hiroki Hamamoto, Keitaro Tanaka (Osaka Medical and Pharmaceutical University, Osaka, Japan); Masanori Yoshimitsu (Hiroshima City North Medical Center Asa Citizens Hospital, Hiroshima, Japan); Hisanaga Horie (Jichi Medical University, Tochigi, Japan); Nobuhisa Matsuhashi (Graduate School of Medicine, Gifu University, Gifu, Japan); Yoshiaki Kuriu (Kyoto Prefectural University of Medicine, Kyoto, Japan); Shuntaro Nagai (Graduate School of Medical Sciences, Kyushu University, Fukuoka, Japan); Madoka Hamada (Kansai Medical University Hospital, Hirakata, Japan); Shinichi Yoshioka (Yao Municipal Hospital, Osaka, Japan); Tamuro Hayama (Teikyo University School of Medicine, Tokyo, Japan); Koki Otsuka (Iwate Medical University School of Medicine, Iwate, Japan); Yusuke Inoue (Nagasaki University, Nagasaki, Japan); Kazuki Ueda (Kindai University Faculty of Medicine, Osaka, Japan); Yuji Toiyama (Mie University, Tsu, Japan); Satoshi Maruyama (Niigata Cancer Center Hospital, Niigata, Japan); Shigeki Yamaguchi (Tokyo Women's Medical University, Tokyo, Japan); Takeshi Naitoh, Masahiko Watanabe (Kitasato University School of Medicine, Kanagawa, Japan).

## Supplementary Material

zrag128_Supplementary_Data

## Data Availability

The data sets generated and/or analysed during the present study are not publicly available, but are available from the corresponding author upon reasonable request.
