# Supplementary material for: Anterior tumour location as a risk factor for local recurrence after laparoscopic surgery for stage I ultra-low rectal cancers: exploratory analysis of the Ultimate trial
Source: BJS Open. 2026 Sep 30;10(5):zrag128. doi: 10.1093/bjsopen/zrag128 (PMC13626249; doi:10.1093/bjsopen/zrag128)
Supplement: Supplementary file 1 [file zrag128_supplementary_data.zip › Supplementary_Material.docx]

**Anterior tumour location as a risk factor for local recurrence after laparoscopic surgery for clinical stage I ultra-low rectal cancers within 5 cm from the anal verge: An exploratory supplemental analysis of the ULTIMATE study**

Atsushi Kohyama^1, 2^, Shinobu Ohnuma^1^, Yuichiro Tsukada^3^, Michiaki Unno^1^, Jun Watanabe⁴^,5^, Yosuke Fukunaga ^6,7^, Yasumitsu Hirano^8^, Kazuhiro Sakamoto^9^, Hiroki Hamamoto^10^, Masanori Yoshimitsu^11,12^, Hisanaga Horie^13^, Nobuhisa Matsuhashi^14^, Yoshiaki Kuriu^15^,Shuntaro Nagai^16^, Madoka Hamada^17^, Shinichi Yoshioka^18^, Tamuro Hayama^19^, Koki Otsuka^20^, Yusuke Inoue^21^, Kazuki Ueda^22^,Yuji Toiyama^23^, Satoshi Maruyama^24^, Shigeki Yamaguchi^25^, Keitaro Tanaka^10,26^, Motoko Suzuki^27^, Toshihiro Misumi^27^, Takeshi Naitoh^28^, Masahiko Watanabe^28^, and Masaaki Ito^3^, Ultimate Trial Group in the Japan Society of Laparoscopic Colorectal Surgery

^1^ Department of Surgery, Tohoku University Graduate School of Medicine, Sendai, Japan

^2^ Department of Surgery, Iwaki City Medical Center, Iwaki, Japan

^3^ Department of Colorectal Surgery, National Cancer Center Hospital East, Kashiwa, Japan

⁴ Department of Surgery, Gastroenterological Center, Yokohama City University Medical Center, Yokohama, Japan

⁵ Department of Colorectal Surgery, Kansai Medical University, Osaka, Japan
⁶ Department of Gastroenterological Surgery, Cancer Institute Hospital, Japanese Foundation of Cancer Research, Tokyo, Japan

^7^ Department of Colorectal Surgery, Kansai Medical University Medical Centre, Osaka, Japan

^8^ Department of Gastroenterological Surgery, Saitama Medical University International Medical Center, Hidaka, Japan

^9^ Department of Coloproctological Surgery, Juntendo University Faculty of Medicine, Tokyo, Japan
^10^ Department of General and Gastroenterological Surgery, Osaka Medical and Pharmaceutical University, Osaka, Japan
^11^ Department of Surgery, Hiroshima City North Medical Center Asa Citizens Hospital, Hiroshima, Japan

^12^ Department of Surgery, Hiroshima City Hiroshima Citizens Hospital, Hiroshima, Japan
^13^ Department of Surgery, Jichi Medical University, Tochigi, Japan
^14^ Department of Gastroenterological Surgery and Pediatric Surgery, Graduate School of Medicine, Gifu University, Gifu, Japan
^15^ Department of Surgery, Kyoto Prefectural University of Medicine, Kyoto, Japan
^16^ Department of Surgery and Oncology, Graduate School of Medical Sciences, Kyushu University, Fukuoka, Japan
^17^ Department of Gastrointestinal Surgery, Kansai Medical University Hospital, Hirakata, Japan
^18^ Department of Surgery, Yao Municipal Hospital, Osaka, Japan
^19^ Department of Surgery, Teikyo University School of Medicine, Itabashi, Japan
^20^ Department of Surgery, Iwate Medical University School of Medicine, Shiwa, Iwate, Japan
^21^ Department of Surgery, Graduate School of Biomedical Sciences, Nagasaki University, Nagasaki, Japan
^22^ Division of Endoscopic & Colorectal Surgery, Department of Surgery, Kindai University Faculty of Medicine, Osaka, Japan
^23^ Department of Gastrointestinal and Pediatric Surgery, Mie University, Tsu, Japan
^24^ Department of Gastroenterological Surgery, Niigata Cancer Center Hospital, Niigata, Japan
^25^ Division of Colorectal Surgery, Department of Surgery, Tokyo Women's Medical University, Tokyo, Japan
^26^ Department of General, Breast and Digestive Surgery, Otsu City Hospital, Otsu, Japan
^27^ Department of Data Science, National Cancer Center Hospital East, Chiba, Japan
^28^ Department of Lower Gastrointestinal Surgery, Kitasato University School of Medicine, Kanagawa, Japan

**Corresponding author.** Atsushi Kohyama, Department of Surgery, Tohoku University School of Medicine

Address: 1-1 Seiryo-machi, Aoba-ku, Sendai 980-8574, Japan. Tel: +81-22-717-7208, Fax: +81-22-717-7209, E-mail: [atsushikohyama@gmail.com](mailto:atsushikohyama@gmail.com). **ORCID ID**: 0000-0001-5807-668X

**Supplementary Materials - Index**

| **Supplementary Figures and Tables** |  |
| --- | --- |
| Supplementary Figure 1. Flow chart showing identification of patients for analysis. | *page 3* |
| Supplementary Figure 2. Institutional differences in local recurrence outcomes | *page 4* |
| Supplementary Table 1. Pathological depth of invasion by tumour location | *page 5* |
|  |  |

**Supplementary Figures and Tables**

Supplementary Figure 1. Flow chart showing identification of patients for analysis.

Supplementary Figure 2. Kaplan–Meier curves of cumulative local recurrence (LR) stratified by institutional patient volume. To address potential limitations inherent to multicentre studies, we examined institutional differences in local recurrence outcomes. No significant differences were observed between high-volume and low-volume centres. Similarly, for anterior tumours, treatment outcomes did not differ across institutions

a. LR for all patients according to institutional volume (high vs low) (*P*=0.342).
b. LR among patients with anterior rectal cancers according to institutional volume (*P*=0.756).

Supplementary Table S1. Comparison of pathological T stage between anterior and non-anterior tumour sites stratified by clinical T stage

| cT-stage |  | pT-stage |  | All (%) |  | Anterior (%) |  | Non-anterior (%) |  | *P** |
| --- | --- | --- | --- | --- | --- | --- | --- | --- | --- | --- |
| cT1 |  | pTis |  | 4 (2.4%) |  | 1 (1.9%) |  | 3 (2.6%) |  | 0.124 |
|  |  | pT1 |  | 135 (79.9%) |  | 40 (75.5%) |  | 95 (81.9%) |  |  |
|  |  | pT2 |  | 25 (14.8%) |  | 12 (22.6%) |  | 13 (11.2%) |  |  |
|  |  | pT3 |  | 5 (3.0%) |  | 0 (0.0%) |  | 5 (4.3%) |  |  |
| cT2 |  | pTis |  | 1 (0.8%) |  | 0 (0.0%) |  | 1 (1.1%) |  | 0.838 |
|  |  | pT1 |  | 22 (17.2%) |  | 8 (20.0%) |  | 14 (15.9%) |  |  |
|  |  | pT2 |  | 80 (62.5%) |  | 25 (62.5%) |  | 55 (62.5%) |  |  |
|  |  | pT3 |  | 25 (19.5%) |  | 7 (17.5%) |  | 18 (20.5%) |  |  |

Values in parentheses are percentages. ^*^Pearson’s chi-square test.

**Comparison of pathological depth of invasion between anterior and posterior lesions**

Because differences in underlying pathological tumour depth could potentially influence local recurrence, we compared pathological depth of invasion between anterior and posterior lesions. No significant differences were observed between the two groups (Supplementary Table 1).
